# Demographic Characteristics and Inflammatory Biomarker Profile in Psoriatic Arthritis Patients with Comorbid Fibromyalgia: A Cross-Sectional Study

**DOI:** 10.3390/medicina61061050

**Published:** 2025-06-06

**Authors:** Marino Paroli, Chiara Gioia, Daniele Accapezzato, Rosalba Caccavale

**Affiliations:** 1 Clinical Immunology Unit, Department of Clinical Immunology, Department of Clinical, Anesthesiologic and Cardiovascular Sciences, Istituto Chirurgico Ortopedico Traumatologico (ICOT), Sapienza University of Rome, 04100 Latina, Italy; rosalba_caccavale@yahoo.it; 2 Internal Medicine Division, Department of Clinical Immunology, Department of Clinical, Anesthesiologic and Cardiovascular Sciences, Policlinico Umberto I University Hospital, Sapienza University of Rome, 00161 Rome, Italy; chiara.gioia@uniroma1.it (C.G.); daniele.accapezzato@uniroma1.it (D.A.)

**Keywords:** psoriatic arthritis, fibromyalgia, serum biomarkers, autoimmunity, comorbidities

## Abstract

*Background and Objectives:* Psoriatic arthritis (PsA) is a chronic rheumatic disease that is frequently associated with fibromyalgia (FM). The coexistence of FM complicates the evaluation of PsA disease activity and the planning of treatment strategies, as the two conditions share many overlapping clinical symptoms. To investigate the contribution of demographic factors and available serum biomarkers of inflammation and autoimmunity in characterizing the heterogeneity among patients meeting the classification criteria for both PsA and FM. *Materials and Methods*: This cross-sectional, single-center study involved 1547 adult patients evaluated between January 2017 and December 2024 who met the CASPAR criteria for PsA. A patient subgroup also met the 2016 ACR criteria for FM. Demographic data, serum inflammatory markers such as C-reactive protein (CRP) and erythrocyte sedimentation rate (ESR), and autoimmunity markers including antinuclear antibodies (ANA), rheumatoid factor (RF), and anti-citrullinated protein antibodies (ACPA) were evaluated. Statistical analyses included chi-square tests, *t*-tests, Mann–Whitney U tests, and multivariate logistic regression to identify independent predictors associated with the coexistence of PsA and FM. *Results:* A total of 254 patients (16.42%) were diagnosed with concomitant FM. Compared to patients with PsA alone, those with concurrent PsA and FM showed significantly lower C-reactive protein (CRP) levels (0.39 ± 0.74 vs. 2.88 ± 12.31 mg/dL; *p* < 0.001) and a higher frequency of antinuclear antibody (ANA) positivity (13.57% vs. 5.78%; *p* < 0.001). No significant differences were observed in rheumatoid factor (RF) or anti-citrullinated protein antibody (ACPA) positivity between the groups. Multivariate logistic regression identified female sex, ANA positivity, CRP levels ≤ 0.5 mg/dL, and elevated body mass index (BMI) as independent predictors of the presence of concomitant FM. *Conclusions*: Patients with concomitant PsA and FM have a distinct demographic and serological profile, suggesting the existence of a clinically significant subgroup within the PsA population. Recognition of these differences may improve diagnostic accuracy and support the development of personalized, non-immunosuppressive therapeutic strategies for this subgroup of patients.

## 1. Introduction

Psoriatic arthritis (PsA) is a chronic, immune-mediated, inflammatory musculoskeletal disorder that occurs in association with psoriasis. It is characterized by a wide and heterogeneous clinical spectrum, which may include peripheral arthritis affecting large and small joints, axial involvement manifesting as inflammatory back pain and sacroiliitis, enthesitis involving inflammation at the sites where tendons or ligaments insert into the bone, and dactylitis [1]. The diagnostic process in PsA can be particularly challenging due to the considerable variability in clinical presentation and the often subtle or nonspecific nature of symptoms, which may evolve over time. This complexity is further heightened when patients primarily report symptoms such as persistent fatigue, non-restorative sleep, or cognitive difficulties (often referred to as ‘fibro fog’), which are hallmark features of fibromyalgia (FM). These overlapping manifestations can obscure the clinical picture, delay the recognition of underlying inflammatory disease, and complicate the differential diagnosis, especially in cases where objective signs of inflammation are minimal or absent [2,3,4,5]. This symptomatic overlap between psoriatic PsA and FM may significantly contribute to misdiagnosis or delayed diagnosis in routine clinical practice. The presence of widespread pain, fatigue, and other non-specific symptoms commonly seen in FM can mask the inflammatory nature of PsA or be erroneously attributed solely to a functional somatic syndrome. As a result, clinicians may underestimate disease activity, overlook subtle signs of inflammation, or initiate inappropriate management strategies, ultimately delaying the implementation of targeted therapies that could prevent joint damage and improve long-term outcomes.

FM is a chronic pain syndrome characterized by a constellation of symptoms that include widespread musculoskeletal pain, persistent fatigue, non-restorative sleep, cognitive disturbances often described as ‘fibro fog’, and frequently comorbid mood disorders such as anxiety and depression. The underlying mechanism of pain in FM is thought to involve central sensitization, whereby altered processing of nociceptive input within the central nervous system leads to pain amplification and a lowered pain threshold in the absence of ongoing peripheral inflammation or tissue damage. Reflecting this distinct pathophysiology, FM has recently been reclassified by the International Association for the Study of Pain (IASP) as a primary chronic pain (PCP) syndrome, a category encompassing conditions in which pain itself is the disease, rather than a symptom of another underlying pathology [6], and its underlying pain mechanism has been defined as nociplastic [7,8]. Beyond chronic widespread pain, FM is frequently associated with a range of comorbid conditions, including irritable bowel syndrome (IBS), migraine, temporomandibular joint disorders (TMJDs), interstitial cystitis, and other functional somatic syndromes. These overlapping disorders further contribute to the symptom burden and functional impairment experienced by patients with FM. Although the precise etiology of FM remains incompletely understood, it is widely accepted to result from a multifactorial interplay involving genetic predisposition, environmental triggers (such as physical or emotional stress), and psychosocial influences. Emerging evidence also suggests a potential role for dysregulated immune responses and low-grade inflammation, raising the possibility that, in some individuals, FM may share pathophysiological features with certain autoimmune or inflammatory conditions [9,10,11].

The coexistence of FM in patients with PsA introduces significant diagnostic and therapeutic complexities [12], particularly because FM-related symptoms can mimic or mask inflammatory activity. This overlap complicates the clinical assessment of PsA disease activity, often making it difficult to distinguish inflammation-driven symptoms from those due to altered central pain processing typical of FM [13,14]. Consequently, FM comorbidity may lead to both overestimation and underestimation of PsA activity, potentially resulting in misclassification of disease severity, inappropriate intensification of immunosuppressive therapy, and poorer treatment outcomes [15,16,17,18].

Given these challenges, there is an urgent need for accessible and reliable biomarkers that can distinguish between active PsA and coexisting FM. These include serum inflammatory markers such as C-reactive protein (CRP) and erythrocyte sedimentation rate (ESR), as well as markers of autoimmunity, including antinuclear antibodies (ANA), rheumatoid factor (RF), and anti-citrullinated protein antibodies (ACPA). Although several inflammatory/autoimmunity biomarkers have been investigated in an attempt to identify those that could help diagnose PsA [19], we focused on those that could be easily accessed in routine clinical practice. Furthermore, these biomarkers differ for pathogenetic reasons from the biomarkers of central pain sensitization present in FM, whose research is still at a very early stage [20].

Determining whether these common laboratory parameters offer adequate diagnostic accuracy can support more precise treatment decisions and help clinicians avoid the risks of overtreatment or undertreatment in these patients with overlapping inflammatory and pain symptoms.

## 2. Methods

This retrospective, single-center study included patients evaluated at our rheumatology clinic between January 2017 and December 2024. Inclusion criteria were compliance with CASPAR (ClASsification criteria for Psoriatic ARthritis) criteria for PsA [21] with or without simultaneous compliance with the 2016 ACR criteria for FM, who had a WPI (Widespread Pain Index) score ≥ 7 and an SSS (Symptom Severity Scale) score ≥ 5, or a WPI of 4–6 with an SSS score ≥ 9 [22]. This study included only adult patients aged 18 years or older. In order to minimize potential confounding variables, we selected individuals who had not received previous rheumatologic treatments and who were on a stable therapeutic regimen at the time of data collection. Demographic data collected comprised gender, age, and body mass index (BMI). Since this was a single-center study, most of the patients were of Caucasian origin, although about 15% were of other ethnic origins, reflecting the current migration flows occurring in European countries.

The laboratory parameters evaluated included antinuclear antibodies (ANA), erythrocyte sedimentation rate (ESR), C-reactive protein (CRP), extractable nuclear antigens (ENA), anti-citrullinated protein antibodies (ACPA), and rheumatoid factor (RF). The reference values used to define normal serological profiles were as follows: ANA titer < 1:80, CRP ≤ 0.5 mg/dL, and negative results for ENA, ACPA, and RF. The ANA test, even when performed using an ELISA test, was always confirmed by indirect immunofluorescence. However, no differentiation was made in the analysis between different fluorescence patterns (e.g., homogeneous and speckled). The cutoff was set at ≥1:80, as specified above.

All procedures were conducted in full accordance with the ethical standards of the Declaration of Helsinki. Prior to analysis, all patient data were anonymized to ensure confidentiality and privacy.

Statistical analyses were performed using GraphPad Prism, version 9.0 (GraphPad Software, San Diego, CA, USA). Categorical variables were compared using the chi-square (χ^2^) test, while continuous variables were analyzed using either Student’s *t*-test or the Mann–Whitney *U* test, depending on the distribution of the data as assessed by the Shapiro–Wilk test. A two-tailed *p*-value of less than 0.05 was considered statistically significant. To identify independent predictors associated with the presence of fibromyalgia features in patients with PsA, a multivariate logistic regression model was employed.

## 3. Results

A total of 1547 patients were included in the analysis, of whom 254 (16.42%) fulfilled both the CASPAR criteria for PsA and the 2016 ACR criteria for FM, constituting the PsA + FM group. The remaining 1293 patients met criteria for PsA only and served as the comparison group. Demographic and clinical characteristics revealed notable differences between the two populations. The PsA + FM group had a significantly higher proportion of female patients compared to the PsA-only group (99.6% vs. 78.81%, *p* < 0.001) and a younger mean age (53.99 ± 11.97 vs. 59.75 ± 14.67 years, *p* < 0.001). Additionally, patients with comorbid FM exhibited a significantly higher body mass index (BMI), with a mean value of 28.69 ± 6.66 compared to 26.86 ± 5.01 in the PsA-only group (*p* < 0.001) (Table 1).

With regard to inflammatory biomarkers, marked differences emerged. The proportion of patients with elevated C-reactive protein (CRP > 0.5 mg/dL) was significantly lower in the PsA + FM group (3.88%) than in those with PsA alone (34.83%, *p* < 0.001). Based on these results, mean CRP ± SD levels were significantly lower in the PsA + FM group (0.39 ± 0.74 mg/dL) with a median of 0.29 mg/dL (IQR: 0.16–0.38 mg/dL) compared to the PsA-only group (2.88 ± 12.31 mg/dL, *p* < 0.001) with a median of 0.39 mg/dL (IQR: 0.21–0.98 mg/dL). ESR values showed a similar difference, with the PsA + FM group showing a lower mean ± SD (18.21 ± 14.39 mm/h) with a median of 13.0 mm/h (IQR: 8.0–24.0 mm/h) compared to the PsA-only group (22.76 ± 23.23 mm/h, *p* < 0.001) with a median of 18.0 mm/h (IQR: 11.0–34.0 mm/h).

When applying a threshold of 25 mm/h as an arbitrary cut-off, the proportion of patients with elevated ESR was 23.26% in the PsA + FM group and 37.76% in the PsA-only group (*p* < 0.001).

**Figure 1:**
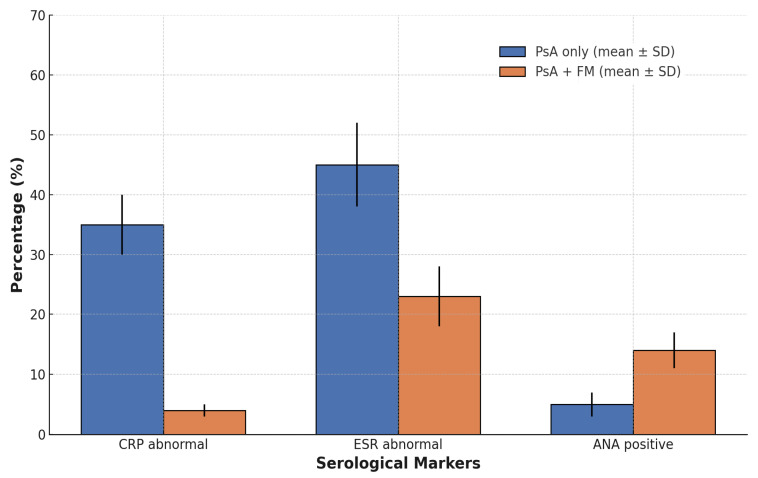
Comparison of serological marker abnormalities between patients with PsA only and those with concomitant FM. Data are presented as mean percentages ± standard deviation. Patients with both PsA and FM exhibited significantly lower rates of CRP and ESR abnormalities but higher ANA positivity, indicating a distinct serological profile. Statistically significant differences were observed for all comparisons (*p* < 0.001).

Serological autoimmune markers also revealed group-specific patterns. Antinuclear antibodies (ANA, titer ≥ 1:80) were significantly more prevalent among patients with FM (13.57%) compared to those with PsA only (5.78%, *p* < 0.001) (Figure 1). In contrast, the prevalence of anti-citrullinated protein antibodies (ACPA) and rheumatoid factor (RF) remained low in both groups and did not differ significantly (ACPA positivity: 0.78% (*n* = 2) in PsA + FM vs. 1.65% (*n* = 21) in PsA-only, *p* = 0.46; RF positivity: 4.26% (*n* = 11) vs. 5.26% (*n* = 68), *p* = 0.43).

**Figure 2:**
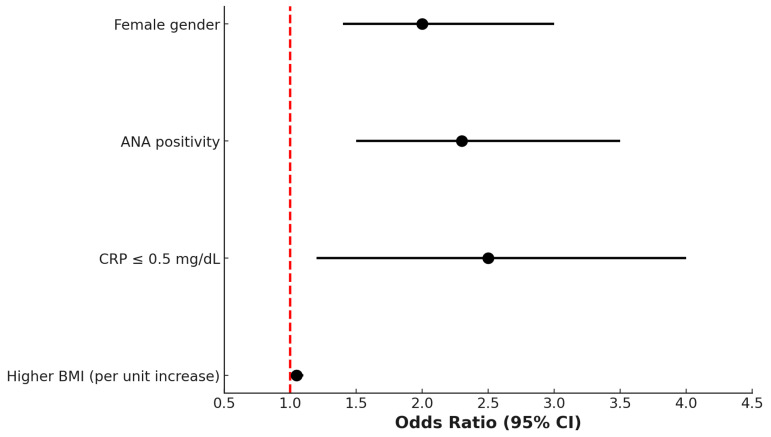
Multivariate logistic regression analysis showing factors independently associated with the coexistence of fibromyalgia (FM) in patients with psoriatic arthritis (PsA). Female gender (OR 2.2, 95% CI 1.6–3.1), antinuclear antibody (ANA) positivity (OR 1.9, 95% CI 1.3–2.8), CRP ≤ 0.5 mg/dL (OR 2.4, 95% CI 1.6–3.7), and higher BMI per unit (OR 1.05, 95% CI 1.02–1.08) were significantly associated with the PsA + FM phenotype. The dashed line represents the null value (OR = 1).

Multivariate logistic regression analysis identified several variables independently associated with the PsA + FM phenotype. Female gender (odds ratio [OR] 2.2; 95% confidence interval [CI]: 1.6–3.1), ANA positivity (OR 1.9; 95% CI: 1.3–2.8), low CRP levels (≤0.5 mg/dL; OR 2.4; 95% CI: 1.6–3.7), and higher BMI (OR 1.05 per unit increase; 95% CI: 1.02–1.08) were all significantly associated with the presence of comorbid FM in patients with PsA (Figure 2).

## 4. Discussion

The primary objective of this study was to investigate whether demographic characteristics and routinely available serological markers of inflammation and autoimmunity could assist clinicians in distinguishing patients who meet the classification criteria for PsA alone from those who also fulfill the criteria for FM. Our findings revealed that 16.42% of patients diagnosed with PsA also met the 2016 ACR criteria for FM, highlighting the substantial prevalence of comorbid FM in this population.

The study spanned from 2017 to 2024, a period during which validated and widely accepted classification criteria—namely the CASPAR criteria for PsA and the 2016 ACR criteria for FM—were consistently applied. This temporal consistency helped ensure diagnostic homogeneity and robustness of case identification across the entire cohort. The observed prevalence aligns with, and slightly exceeds, prior reports in the literature, which have estimated the frequency of FM among PsA patients to range between 10% and 14% [16,23]. This finding reinforces the clinical relevance of FM as a frequent and potentially underrecognized comorbidity in patients with PsA.

The presence of FM in PsA patients may influence symptom perception, disease activity scores, and treatment outcomes, thus posing significant diagnostic and therapeutic challenges. In this context, the identification of simple clinical or laboratory parameters that may help discriminate between inflammatory and non-inflammatory sources of pain is of particular interest and clinical utility [24,25,26]. The frequent association of FM with a wide range of both rheumatic and non-rheumatic conditions has been well documented in the literature. FM commonly coexists with autoimmune inflammatory diseases such as rheumatoid arthritis, systemic lupus erythematosus, and PsA, but it is also prevalent among individuals with non-inflammatory disorders, including irritable bowel syndrome, migraine, and temporomandibular joint disorders. This tendency toward comorbidity underscores the complex and multifactorial nature of FM and highlights its potential to complicate the clinical assessment of coexisting diseases, particularly those in which subjective symptoms such as pain and fatigue already play a central role [27].

A strong association between female sex and the presence of FM in patients with PsA emerged from our analysis, with women significantly overrepresented in the PsA + FM group. This finding is consistent with a well-established body of evidence indicating a pronounced female predominance in FM prevalence across diverse rheumatologic populations. Hormonal, neurobiological, and psychosocial factors have been proposed to contribute to this sex-based susceptibility, although the precise mechanisms remain incompletely understood. In the context of PsA, this observation reinforces the importance of adopting a sex-sensitive approach when evaluating pain, fatigue, and disease activity, particularly in female patients presenting with complex symptom profiles [28]. The PsA + FM group was composed of 99.6% women. This percentage far exceeds the typical gender ratio in FM (~80–90% women) [29]. The high percentage of women may reflect a referral bias in our center, which may attract a higher percentage of female patients due to healthcare-seeking behaviors or referral practices. This result, which effectively excludes the percentage of men affected by fibromyalgia and whose characteristics may differ from those of women, could partly influence the interpretation of the results. Therefore, studies including a larger number of men are needed to confirm the results of the present study.

The odds ratio (OR) of 2.2 identified in our cohort further substantiates the significant association between female sex and the presence of comorbid FM in patients with psoriatic PsA and supports the hypothesis that a combination of biological, hormonal, and psychosocial factors contributes to the heightened susceptibility of women to FM. Estrogen-mediated modulation of pain processing, differences in stress response systems, and greater exposure to psychosocial stressors have all been implicated as potential mechanisms underlying this sex-related vulnerability. These findings emphasize the need for heightened clinical awareness when evaluating female PsA patients, particularly in the presence of diffuse pain, fatigue, and other non-specific symptoms that may overlap with FM [30,31,32].

Patients with concomitant PsA and FM were also found to be significantly younger than those with PsA alone. While the age difference reached statistical significance, its clinical relevance may be considered modest. Nonetheless, this observation is in line with previous studies suggesting that FM-related symptoms may exert a more profound impact on younger individuals affected by inflammatory arthritis. Several hypotheses have been proposed to explain this phenomenon, including age-related differences in central pain processing, emotional resilience, and coping strategies. Younger patients may exhibit heightened pain sensitivity or greater psychological distress in response to chronic symptoms, which could amplify the subjective burden of disease and complicate the clinical picture. These considerations further highlight the importance of a nuanced, individualized approach to symptom assessment in younger PsA patients presenting with features suggestive of FM [33,34,35]. Furthermore, higher body mass index (BMI) values were observed among patients with concomitant PsA and FM compared to those with PsA alone. This finding is consistent with existing literature suggesting that obesity independently increases the risk of developing both psoriatic arthritis and fibromyalgia. In PsA, excess adiposity has been linked to heightened systemic inflammation, mechanical stress on joints, and reduced treatment responsiveness, while in FM, obesity has been associated with increased pain severity, sleep disturbances, and reduced physical functioning. The coexistence of these two conditions in individuals with elevated BMI may reflect the compounding effects of metabolic dysregulation, pro-inflammatory cytokine activity, and altered pain perception pathways. These data underscore the importance of addressing weight management as part of a comprehensive therapeutic strategy in this complex patient population [36,37]. Obesity may exacerbate systemic inflammation through increased production of adipokines and pro-inflammatory cytokines, such as TNF-α and IL-6, which have been suggested to play a role in the pathogenesis of either PsA or FM [38,39]. Additionally, excess body weight imposes mechanical stress on joints and entheses, potentially aggravating musculoskeletal symptoms and functional impairment [40]. Emerging evidence also suggests that obesity can influence central pain modulation by altering neurotransmitter systems and increasing pain sensitivity. A large study found that central obesity was linked to an increased risk of multisite pain even after taking demographic and health factors into account [41]. Together, these mechanisms offer a biologically and clinically plausible explanation for the observed association between higher BMI and the coexistence of PsA and FM.

From a serological perspective, one of the most noteworthy findings was the significantly lower CRP levels observed in patients with concomitant FM. CRP is a well-established biomarker of systemic inflammation and synovitis, and it is routinely incorporated into composite disease activity indices for PsA, such as the Disease Activity Index for Psoriatic Arthritis (DAPSA) and the Psoriatic Arthritis Disease Activity Score (PASDAS). The markedly lower CRP levels in PsA + FM patients suggest that, in many cases, the clinical burden reported by these individuals may not be driven by active inflammatory processes. This discrepancy highlights the risk of overestimating disease activity based solely on patient-reported outcomes in the absence of objective inflammatory markers and underscores the importance of integrating laboratory data into the clinical assessment to guide therapeutic decision-making. These findings also support the use of CRP as a potentially useful negative predictor of comorbid FM in patients with PsA [42,43]. The observed reduction in CRP among PsA patients with comorbid FM suggests a clinical profile in which central sensitization mechanisms may predominate over active peripheral inflammation. In such cases, the heightened symptom burden—characterized by widespread pain, fatigue, and cognitive complaints—is likely not reflective of ongoing synovial or entheseal inflammation but rather of dysregulated central pain processing. These findings align with previous studies reporting that FM comorbidity is associated with lower levels of objective inflammatory biomarkers, despite paradoxically higher patient-reported outcomes and disease activity scores. This discordance underscores the diagnostic complexity posed by FM in the context of PsA and highlights the limitations of relying solely on subjective measures when assessing disease activity in this population. In our study, we observed a wide variability in C-reactive protein (CRP) levels in the PsA-only group. This suggests the presence of outliers, which could potentially influence the statistical analysis. Extreme CRP values (>10 mg/dL) were retained in the analysis to reflect the clinical reality of our cohort. However, we conducted a sensitivity analysis after winsorizing these values and found no significant differences in the main results, confirming the robustness of our findings.

In addition, it is important to note that CRP elevation is not universally present in active PsA, which somewhat limits its sensitivity as a standalone marker of disease activity. A substantial proportion of PsA patients may exhibit clinically evident synovitis or axial inflammation despite normal CRP levels, particularly in cases with predominant enthesitis or oligoarticular involvement. Therefore, while low CRP values in PsA patients with comorbid FM may suggest a non-inflammatory pain phenotype, normal CRP cannot definitively exclude active PsA [44]. This nuance reinforces the need for a comprehensive clinical evaluation, incorporating physical examination findings, imaging modalities when appropriate, and longitudinal assessment of symptom patterns to accurately interpret laboratory data in the diagnostic workup.

Some studies have reported increased levels of pro-inflammatory cytokines in the serum of patients with FM [45]. The observed lower CRP levels in PsA + FM patients may be explained by the fact that CRP, as a downstream marker of systemic inflammation, may not adequately reflect subtle or localized inflammatory pathways relevant in FM.

Additionally, the higher prevalence of antinuclear antibody (ANA) positivity among patients with concomitant PsA and FM, compared to those with PsA alone, warrants careful consideration. Although ANA positivity in our cohort was generally low and frequently detected at low titers—levels often considered clinically nonspecific—its increased frequency in the PsA + FM group may point to a heightened state of immune system activation or an underlying predisposition to broader autoimmune reactivity within this subset of patients. This observation is consistent with previous studies reporting elevated ANA prevalence among individuals with FM, regardless of the presence of inflammatory rheumatic disease. The possible autoimmune nature of fibromyalgia has been recently suggested by an elegant study showing that IgG antibodies from patients with FM can induce sensory hypersensitivity in mice by sensitizing peripheral nociceptive neurons. In contrast, IgG-depleted serum had no effect. The study also revealed that FM IgG targets satellite glial cells, neurons, and some macrophages in dorsal root ganglia but not spinal cord cells, proposing that therapies targeting IgG may be beneficial for treating FM [9]. However, the clinical significance of ANA positivity in FM remains a matter of ongoing debate, as low-titer ANAs are also found in a portion of the healthy population and in various non-autoimmune conditions. Despite this, their presence in PsA patients with FM may contribute to diagnostic uncertainty and may lead to more extensive investigations or misclassification in routine clinical practice [46,47]. In addition, periodic clinical evaluation is recommended to monitor for the development of autoimmune features in patients with positive ANA [48]. Conversely, the rates of ACPA and RF positivity remained comparably low in both the PsA + FM and PsA-only groups, reaffirming the limited diagnostic utility of these serological markers in distinguishing between these two subpopulations. This finding is consistent with their established role as highly specific biomarkers for rheumatoid arthritis (RA), rather than for PsA, and supports the notion that ACPA and RF are not characteristic features of PsA regardless of the presence of fibromyalgia. Their low prevalence in both groups further underscores the importance of not relying on these markers when attempting to differentiate PsA from other inflammatory arthritides or from conditions characterized by chronic widespread pain, such as FM [49]. Multivariate logistic regression analysis confirmed that female sex, elevated body mass index (BMI), ANA positivity, and low CRP levels were independently associated with the presence of concomitant FM in patients with PsA. Taken together, these findings delineate a distinct clinical and serological profile that may aid in identifying PsA patients at higher risk for FM comorbidity. This distinction is of particular clinical relevance, as it underscores the importance of a nuanced, individualized diagnostic approach that goes beyond the assessment of inflammatory activity alone and incorporates both subjective symptoms and objective laboratory findings. Recognizing this phenotype may help clinicians avoid diagnostic delay, misclassification, or inappropriate treatment escalation in patients whose symptom burden is not driven by active inflammation. Furthermore, it is important to emphasize the importance of integrated therapeutic approaches for patients identified as being at high risk of developing FM in the context of PsA. Clinicians should consider implementing comprehensive pain management strategies, patient education initiatives, and multidisciplinary care pathways that address both musculoskeletal inflammation and centralized pain mechanisms. In addition, patients identified as being at high risk of FM should undergo close clinical monitoring, with a focus on the evolution of pain symptoms and the need for personalized management interventions. This approach aims to optimize patient outcomes by addressing the unique challenges posed by the overlap of PsA-FM and ensuring that emerging pain-related issues are identified and managed promptly.

Among the primary strengths of this study are the relatively large sample size and the consistent application of validated classification criteria, namely the CASPAR criteria for PsA and the 2016 ACR criteria for FM, across a multi-year study period. Furthermore, the systematic assessment of both demographic and serological variables allowed for a comprehensive evaluation of potential distinguishing features between PsA patients with and without FM. Importantly, by excluding patients receiving active rheumatologic treatment, we were able to reduce confounding from therapy-induced changes in inflammatory biomarkers, thereby strengthening the internal validity of the observed associations. Furthermore, since our study included untreated or stable patients, it was possible to exclude the potential confounding effect of active disease or frequent flare-ups, as these conditions could influence pain perception, fatigue, and inflammatory markers, thereby affecting the study results.

However, several limitations must be acknowledged. First, the retrospective nature of the study design and the fact that this was a single-center study introduce the potential for selection and information biases and limit the ability to draw causal inferences. Second, although standardized classification criteria were employed, the diagnosis of FM remains inherently subjective and may be influenced by interobserver variability. Third, the exclusion of patients undergoing active treatment may restrict the generalizability of our findings to broader PsA populations, particularly those with more severe disease or those currently managed with biologic or targeted synthetic DMARDs. Fourth, since this was a single-center study in Italy, the majority of the patients were of Caucasian origin, which may not reflect the diversity of ethnicities. It is worth noting that the incidence of PsA and FM varies between Nordic and Mediterranean countries of Europe [50,51]. To address this concern, future multicenter studies involving more diverse populations are needed to validate our findings in different ethnic and genetic settings. Finally, the absence of data on psychological comorbidities, such as anxiety and depression, which are known to modulate FM symptomatology, may have resulted in residual confounding, and future studies should aim to integrate these variables into the analysis. It should also be emphasized that the detection of a very low number of rare events, such as positivity for ACPA or RF, could represent a type II error due to the sample being too small to detect these differences.

In conclusion, this study highlights the clinical and laboratory features that characterize PsA patients with comorbid FM and supports the utility of routine biomarkers and demographic profiling in improving diagnostic precision. Future prospective studies incorporating imaging, patient-reported outcomes, and psychological assessments will be crucial to further refine the diagnostic approach and optimize care for this complex subgroup of patients.

Recent evidence suggests that musculoskeletal ultrasound (US) assessment of joints, tendons, and entheses may outperform conventional composite clinical scores in evaluating disease activity among PsA patients with comorbid FM. Given that FM can inflate subjective components of disease activity indices, such as patient global assessment and pain scores, US offers an objective and sensitive tool for detecting active inflammation, particularly in cases where clinical evaluation is equivocal or potentially confounded by central sensitization. Incorporating ultrasound into routine assessment may therefore enhance diagnostic precision, guide therapeutic decisions more accurately, and help avoid both overtreatment of non-inflammatory symptoms and undertreatment of true inflammatory disease. Future prospective studies should explore the integration of imaging modalities such as US into composite indices specifically tailored for PsA populations with high symptom burden and FM overlap [52].

## 5. Conclusions

This study highlights that female sex, elevated body mass index (BMI), ANA positivity, and low C-reactive protein (CRP) levels are independently associated with the presence of FM features in patients with PsA. These routinely accessible demographic and serological parameters, when interpreted alongside a thorough clinical assessment, may assist clinicians in identifying PsA patients with comorbid FM, thereby enhancing diagnostic accuracy and informing more individualized treatment strategies. It is important to note that CRP levels must be interpreted in conjunction with clinical assessment, as they alone do not provide sufficient specificity to serve as a stand-alone diagnostic marker. Longitudinal studies evaluating changes in CRP and BMI after FM diagnosis are recommended to clarify causal relationships.

Future studies that integrate imaging modalities, such as musculoskeletal ultrasound, biomarkers of central sensitization, and patient-reported outcome measures could offer a more comprehensive understanding of this complex clinical intersection. Additionally, interventional trials aimed at evaluating targeted management approaches for this subgroup are essential to optimize care and improve patient outcomes.

## Figures and Tables

**Table 1:** Demographic characteristics of PsA patients with and without fibromyalgia.

Characteristic	PsA + FM (*n* = 254)	PsA only (*n* = 1293)	*p*-Value	Notes
Female, %	99.6%	78.81%	<0.001	Significantly higher in PsA + FM
Mean age, years (mean ± SD)	53.99 ± 11.97	59.75 ± 14.67	<0.001	Significantly lower in PsA + FM
BMI (mean ± SD)	28.69 ± 6.66	26.86 ± 5.01	<0.001	Significantly higher in PsA + FM

## Data Availability

The data presented in this study are available on request from the corresponding author due to privacy reasons.
